# Genomic inbreeding and selection signatures analyses in the Doberman Pinscher breed

**DOI:** 10.1371/journal.pone.0351912

**Published:** 2026-10-08

**Authors:** Henrique Mulim-McCarthy, Breno Fragomeni, Sophie Liu, Hinayah Rojas de Oliveira

**Affiliations:** 1 Department of Animal Sciences, Purdue University, West Lafayette, Indiana, United States of America; 2 Department of Animal Science, University of Connecticut, Storrs, Connecticut, United States of America; 3 Doberman Diversity Project, Oakland, California, United States of America; West Bengal University of Animal and Fishery Sciences, INDIA

## Abstract

The Doberman Pinscher population has undergone strong artificial selection for morphology and behavior, which can reduce genomic diversity and increase autozygosity. Here, we characterized the genome structure and identified selection signatures in Doberman Pinschers using complementary within- and between-population approaches. Genotypes from 3,226 Dobermans Dogs (Illumina CanineHD; 216,184 SNPs) provided by the Doberman Diversity Project were analyzed after purpose-specific quality control. Genomic inbreeding was quantified using four allele-frequency–based metrics and the runs of homozygosity (F_ROH_) approach. Selection signatures were detected using intrapopulation (i.e., Runs of Homozygosity—ROH; Integrated Haplotype Score—iHS; and Number of Segregating Sites by Length—nSL) and interpopulation methods (i.e., Fixation Index—FST; Cross-Population Extended Haplotype Homozygosity—XP-EHH; and Cross-Population Number of Segregating Sites by Length—XP-nSL) comparing the Doberman Pinscher breed to Labrador Retriever (n=237). Dobermans showed high overall inbreeding, with a mean F_ROH_ of 0.42 (range 0.22–0.68), whereas the allele-frequency–based inbreeding estimators had similar means (~0.04). The partitioning of the ROH indicated high contributions from medium-to-long ROHs, consistent with recent inbreeding. The ROH scans identified 39,512 SNPs in ROH islands (≥50% frequency across individuals), with notable concentrations on CFA2, CFA3, and CFA31. Haplotype-based scans identified 2,820 candidate iHS SNPs and 2,173 candidate nSL SNPs (|score|>2). A common set of 310 SNPs was shared among ROH, iHS, and nSL, mapping near 279 genes that were mostly enriched for developmental pathways, particularly neurodevelopment and neuron-related cellular components. Between breeds, 349 highly differentiated SNPs were detected by FST, while XP-EHH and XP-nSL highlighted more than 1,000 haplotype-based signals differentiating Doberman Pinschers from the Labrador Retriever reference population. A total of nine SNPs overlapped across FST, XP-EHH, and XP-nSL, which were located mainly on CFA8 (~59.48–60.61 Mb) near the *KCNK10*, *SPATA7*, *PTPN21, NEGR1,* and BTG1 genes. Functional annotations for some candidate genes, including BTG1 and *KCNK10*, suggest possible links to cellular regulatory and cardiac-related biological processes; however, direct associations with Doberman disease phenotypes remain to be validated. Overall, the Doberman Pinscher breed exhibits high genome-wide autozygosity and levels of inbreeding. In addition, our results showed consistent, multi-method evidence of selection at loci associated with neurodevelopmental and regulatory pathways. These findings provide prioritized candidate regions for follow-up studies that integrate phenotypes relevant to breed health and performance.

## Introduction

The Doberman Pinscher breed originated in the late 19th-century, in Germany, where Karl Friedrich Louis Dobermann, a tax collector, began developing the breed to serve as a loyal, protective companion and an effective guard dog during his rounds [1]. To achieve this, he crossed several breeds, including Rottweiler, Greyhound, and German Pinscher, producing a dog that combined agility with power [2]. Early in its history, the Doberman Pinscher became recognized for its intelligence, loyalty, and protective behavior, and it was widely adopted for working roles, including police and military services. The breed was formally registered with the American Kennel Club (AKC) in 1943, which further established its prominence as both a companion and working dog in the United States [1,2]

In terms of physical appearance, the Doberman Pinscher is a medium-to-large working breed with a lean, muscular, athletic build and a square, well-balanced outline (i.e., its body length is typically close to its height at the withers; [3]). The head is long, narrow, and wedge-shaped, and the coat is short, sleek, and close to the body. Common coat colors include black with rust markings, red (brown) with rust markings, blue with rust markings, and fawn with rust markings [4]. Overall, the breed’s posture and expression are generally described as alert, confident, and intelligent [5].

The strong and direct artificial selection over time has enabled the fixation of desirable morphological and behavioral traits across various dog breeds, making them more predictable in type and temperament [6]. These same breeding practices, however, can increase homozygosity not only for desirable alleles but also for harmful recessive variants, thereby contributing to the emergence and/or elevated frequency of inherited disorders within particular breed populations [7]. Accordingly, the careful management of genomic diversity is central to long-term genetic sustainability in dog breeds. Maintaining sufficient genetic diversity supports health, adaptability, and population viability, while also reducing the risks of inbreeding depression and breed-associated genetic diseases [8,9].

Characterizing the genomic consequences of such selective pressures requires analytical approaches capable of detecting selection footprints across the genome. Selection signature analysis is one of these approaches, designed to identify genomic regions influenced by natural or artificial selection through the detection of specific patterns such as extended regions with reduced genetic variation, long shared haplotypes, and marked differences in allele frequencies between populations or breeds [10,11]. In the context of a highly selected breed such as the Doberman Pinscher, such analyses can provide a deep understanding of genetic diversity and selection history, revealing regions of the genome that have been selected and exhibit reduced diversity.

The objectives of this study were to: (1) characterize the genomic structure of the Doberman Pinscher population; (2) detect selection signatures using three intrapopulation methods (i.e., Runs of Homozygosity—ROH; Integrated Haplotype Score—iHS; and Number of Segregating Sites by Length—nSL) and three interpopulation methods (i.e., Fixation Index—FST; Cross-Population Extended Haplotype Homozygosity—XP-EHH; and Cross-Population Number of Segregating Sites by Length—XP-nSL); and (3) perform gene annotation and functional analyses to identify candidate genes and metabolic pathways linked to genomic regions found in shared loci across the different selection-signature approaches.

## Materials and methods

### Genotypic information

Genotypic information from 3,226 animals (1,489 males and 1,737 females), genotyped with the Illumina Canine HD genotyping array containing 216,184 SNP markers, was used in this study. The genotypic data were initially collected through routine genetic testing of collaborating dogs with the owners’ consent for the Doberman Diversity Project (https://www.dobermandiversityproject.org/), which were made available for this project. Therefore, no animal care committee approval was necessary for this study, as all information required for this study was obtained from existing databases. The dataset was not restricted to a single geographic region. The genotyped dogs represented a global cohort, with most samples from North America and Europe, including the United Kingdom, and a smaller number of samples from Asia, Australia, South America, and some African countries. However, detailed geographic and breeding-purpose metadata were not available for all individuals included in the present analysis, as described by Wade et al. [1].

### Quality control

Different quality control procedures were used depending on the purpose of the analysis. For the analyses in which fixated markers were important (i.e., ROH, XP-EHH, and XP-nSL), a call-rate for animals and genotypes < 0.01 was used, and non-autosomal markers or markers without genomic position were removed. For the analyses that consider only segregating sites (i.e., FST, iHS, nSL), in addition to the initial quality control, markers with minor allele frequency (MAF) < 0.05 and deviating from Hardy-Weinberg equilibrium at the 10^−6^ level were also excluded from the dataset. In the end, 3,226 animals and 190,293 markers were used in the first set of analyses, and 3,226 animals and 61,910 markers were used for the second set of analyses.

### Intrapopulation analyses

#### Population structure and linkage disequilibrium.

Population structure was evaluated using principal component analysis (PCA) implemented in PLINK v1.9 [12]. The PCA was computed from genome-wide SNP data using a variance-standardized genomic relationship structure, in which SNP covariances were scaled by their respective variances to avoid undue influence of markers with different allele-frequency variances.

Genome-wide linkage disequilibrium (LD) was estimated in PLINK software v1.9 [12] as the squared correlation (r2) between alleles at pairs of loci. To characterize LD decay with physical distance, marker pairs were grouped into distance bins, and mean r2 was calculated per bin: 10–100 kb using 10 kb intervals, and 100–1,000 kb using 100 kb intervals. Bins containing fewer than 50 SNP pairs were excluded to ensure stable mean LD estimates. The LD metric was computed using the PLINK options --r2 dprime-signed with-freqs --ld-window 100000 --ld-window-r2 0, which also outputs signed 𝐷′ and allele frequencies for comprehensive evaluation of LD patterns.

#### Inbreeding metrics.

Five different inbreeding metrics were estimated using the genomic information. The first metric applied in this study was based on the additive genetic relationship (F_GRM_ – [13]), derived as:


$$\begin{array}{c} F_{GRM}=G_{ii}-\text{1} \end{array}$$

where


$$\begin{array}{c} G_{ii}=\frac{ZZ\prime }{\sum_{j=i}^{M}\text{2}p_{i}(\text{1}-p_{i})}, \end{array}$$

**G**_**ii**_ is the genomic relationship matrix, **Z** is the nxM matrix of centered genotypes for n individuals and M markers, and p_i_ is the reference allele frequency in the population. The second method used in this study was based on the homozygous genotypes observed and expected (F_HOM1_ – [14]):


$$\begin{array}{c} F_{HOM\text{1}}=\frac{H_{exp}-H_{obs}}{m-H_{exp}} \end{array}$$

where H_exp_ is the expected value for homozygous genotypes, H_obs_ is the observed value for the homozygous genotypes, and m is the number of non-missing autosomal loci for each individual. The third method used was based on the excess of homozygosity, which was assessed by computing the mean per-SNP terms ratio instead of the ratios of sums (F_HOM2_ – Yang et al., 2011), i.e.,:


$$\begin{array}{c} F_{HOM\text{2}}=\text{1}-\frac{\text{1}}{s}\sum_{i=\text{1}}^{S}\frac{x_{i}(\text{2}-x_{i})}{{\text{2}p}_{i}(\text{1}-p_{i})} \end{array}$$

where xi ∈ {0,1,2} is the minor-allele count for the individual at SNP i, p_i_ is the minor allele frequency, and S is the number of SNPs.

As the three previous metrics strongly depend on allele frequency, we also tested another metric that reduces the impact of allele frequency on the estimate. This metric is based on the correlation between uniting gametes (F_UNI_ - [15]), a direct estimation based on Wright’s definition of inbreeding [16]:


$$\begin{array}{c} F_{UNI}=\frac{\text{1}}{s}\sum_{i=\text{1}}^{S}\frac{x_{i}^{\text{2}}-(\text{1}+\text{2}p_{i})p_{i}+\text{2}p_{i}^{\text{2}}}{{\text{2}p}_{i}(\text{1}-p_{i})} \end{array}$$

where x_i_ is the number of the reference allele copies of the i^th^ SNP, and p_i_ is the reference allele frequency in the population. The final metric used in this study was based on the sum of the individual lengths of ROH divided by the total length of the autosomal genome (F_ROH_ – [17]):


$$\begin{array}{c} F_{ROH}=\frac{\sum_{i=i}^{n}f({ROH}_{i})}{\sum_{j=i}^{A}h(j)} \end{array}$$

where (ROHi) is the total ROH length for individual i, n is the number of homozygous genomic regions in that individual, h(j) is the length of chromosome j, and A is the number of autosomal chromosomes (A = 38). In addition, we calculated inbreeding within each ROH length class (<2 Mb, 2–4 Mb, 4–8 Mb, 8–16 Mb, and >16 Mb) as the sum of ROH lengths in that class divided by the total length of the autosomal dog genome. The first four genomic inbreeding coefficients were computed using PLINK v1.9 [12] while the last method was computed using an in-house R script. Pairwise relationships among genomic inbreeding coefficients were evaluated using scatterplot matrices and correlation analyses. Pearson correlations were used to assess linear relationships among estimators, whereas Spearman correlations were used to evaluate rank-based associations and assess the robustness of the results to potential skewness or extreme observations. Pearson and Spearman correlation heatmaps are provided in the S1 Fig.

#### Runs of homozygosity.

Runs of homozygosity were detected based on the windows approach using the PLINK v 1.9 software [12]. To reduce spurious ROH calls due to random homozygosity, we set the minimum number of markers required to define a ROH using the approach proposed by Lencz et al. [18] where the minimum number of markers (n_min_) is calculated as:


$$\begin{array}{c} n_{min}=\frac{{\text{log}}_{\text{1}\text{0}}\left(\alpha /(nxm\right)}{{\text{log}}_{\text{1}\text{0}}\left(\text{1}-H_{o}\right)} \end{array}$$

where α is the probability of observing a ROH by chance (set to 0.05), *n* is the number of individuals (3,226), *m* is the number of markers (190,293), and *Ho* is the population's mean observed heterozygosity (0.32). Therefore, the minimum number of markers to define a ROH was 61 (--homozyg-snp 61). We further required ROH to be at least 500 kb long (--homozyg-kb 500), with a maximum inter-SNP density of 50 kb/SNP (--homozyg-density 50) and allowing gaps up to 200 kb (--homozyg-gap 200). To account for occasional genotyping errors, we allowed up to 1 heterozygous call within a ROH (--homozyg-het 1). The ROH were scanned using windows of 100 SNPs (--homozyg-window-snp 100), allowing up to 1 missing genotype per window (--homozyg-window-missing 1), and requiring at least 95% of overlapping windows supporting homozygosity (--homozyg-window-threshold 0.05). After identifying the ROHs, the segments were summarized in accordance with the length of the ROH and classified into five classes (<2, 2–4, 4–8, 8–16, and > 16Mb). Markers found in at least 50% of the cases inside of a ROH were considered candidate ROH islands for downstream annotation.

#### Integrated haplotype-based selection scans.

Recent and ongoing positive selection was investigated using the integrated haplotype score (iHS) and number of segregating sites by length (nSL) statistics, which detect unusually long haplotypes associated with a given allele compared with the genome-wide expectation. Analyses were performed on phased haplotypes and run separately for each autosome using the software selscan v3.0.1 [19], then combined into genome-wide result files. The phased dataset used for iHS and nSL analyses included 3,226 animals and 61,910 SNPs, ensuring sufficient marker density and minimizing potential artifacts from low-quality or rare variants. Because a high-resolution genetic map was not available for all markers, we generated a proxy recombination map by assigning genetic positions under a constant recombination rate of 1 cM per Mb, using physical base-pair coordinates to derive genetic distance along each chromosome. The iHS and nSL were computed per chromosome using selscan [19], and the raw outputs were subsequently standardized (normalized) using the selscan norm utility with 20 allele-frequency bins to account for the dependence of these statistics on allele frequency. Candidate selection signals were defined as SNPs with absolute normalized iHS and nSL values greater than 2 [20–22], and these markers were retained for downstream visualization and annotation.

### Interpopulation analyses

#### Quality control in the Labrador population.

For the interpopulation comparisons, we used data from 237 Labrador Retrievers genotyped with 166,171 SNP markers made public available by Campbell et al. [23]. After quality control and restricting to SNPs shared with the Doberman dataset, we generated two working datasets: one for fixation index (FST) analyses containing 47,688 common SNPs, and a second for cross-population (XP) analyses containing 144,855 common SNPs. The MAF and HWE filters were applied for the FST dataset because FST is estimated directly from allele-frequency differences, i.e., rare variants and markers with strong HWE deviations can produce unstable frequency estimates and inflate differentiation due to genotyping artifacts. In contrast, the XP analyses are haplotype-based and were performed on phased data; therefore, we did not additionally filter variants based on HWE for XP beyond the general genotype-level quality control and the requirement that variants are segregating.

We characterized the Labrador Retriever reference population used for between-population analyses, using exploratory analyses included as S2 Figs. These analyses included principal component analysis, LD decay, observed and expected heterozygosity, genomic inbreeding coefficients, pairwise correlations among inbreeding estimators, ROH distribution by chromosome and length class, and ROH islands. Because individual-level metadata regarding working versus show type, coat color, or geographic origin were not available, subgroup-specific analyses could not be performed within the Labrador Retriever. For ROH analyses the same general commands and parameters used for the Doberman Pinscher population were applied, except for the minimum number of SNPs required to define a ROH segment. Based on the observed heterozygosity of the Labrador Retriever reference population, this threshold was set to 46 SNPs.

#### Fixation index.

To reduce redundancy of SNP markers due to LD prior to estimating genetic differentiation, LD pruning was performed in PLINK v 1.9 [12] using a sliding-window approach (--indep-pairwise 50 5 0.8). This procedure evaluates LD within windows of 50 SNPs, shifts the window by 5 SNPs, and removes variants exceeding an LD threshold of r² = 0.8. Only SNPs retained after pruning were used in the dataset for the F_ST_ analysis (n = 47,688). Genetic differentiation between the defined populations was quantified using Wright’s fixation index (F_ST_) as implemented in PLINK v 1.9 [12]:


$$\begin{array}{c} F_{ST}=\frac{a}{a+b+c} \end{array}$$

where *a* is the allele frequency variation among populations, *b* is the sampling variance, and *c* is the heterozygosity within populations. Markers with F_ST_ ≥ 0.80 were considered highly differentiated and were extracted for follow-up annotation.

#### Cross-population haplotype tests.

To detect breed-specific recent selection, we performed cross-population haplotype scans using the selscan software [19], comparing Doberman (target population) against Labrador (reference population). Genotypes were phased per chromosome and population with the BEAGLE software [24], and phased VCFs files were used as direct inputs to selscan. The XP-EHH metric contrasts the integrated decay of extended haplotype homozygosity between populations and can be summarized as:


$$\begin{array}{c} EHH=\text{ln}(\frac{{IHH}_{Doberman}}{{IHH}_{Labrador}}), \end{array}$$

where IHH is the integrated haplotype homozygosity for each population, summarizing how long haplotypes remain homozygous (shared) around that marker. Thus, positive scores indicate unusually long, high-frequency haplotypes in Doberman relative to Labrador, while negative scores indicate stronger signals in Labrador.

Regarding the XP-nSL metric, the estimates will provide a complementary cross-population haplotype statistic, which is sensitive to sweeps across a range of allele frequencies. We quantified recent positive selection using the number of segregating sites by length (nSL) statistic and its cross-population extension (XP-nSL) as implemented in selscan [19]. The nSL is an EHH-based measure of haplotype length that replaces genetic distance with distance measured in numbers of SNPs, reducing sensitivity to variation in local recombination rates. The nSL score at the core site is then defined as the log-ratio of the two allele-specific integrals:


$$\begin{array}{c} nSL=\text{ln}(\frac{{SL}_{Doberman}}{{SL}_{Labrador}}), \end{array}$$

where *SL* is the allele-specific integrated haplotype homozygosity measured in SNP units. Raw XP-EHH and XP-nSL values were normalized with the selscan norm program using 20 bins to obtain standardized genome-wide scores for downstream thresholding and peak detection. Markers with absolute normalized XP-EHH and XP-nSL values ≥ 2 (i.e., |score| ≥ 2) were retained as candidate signals and were subsequently prioritized for downstream functional annotation.

### Functional annotation

To biologically interpret candidate selection signals, we prioritized loci supported by multiple complementary approaches. Specifically, we defined two candidate marker sets: (i) SNPs shared among the intrapopulation analyses (ROH, iHS, and nSL), and (ii) SNPs shared between the interpopulation differentiation and cross-population haplotype tests (FST and XP statistics). These intersecting marker sets were carried forward for functional characterization, focusing on regions consistently identified across methods.

Functional annotation was performed in R using the GALLO package [25], version 2.0. Gene models were obtained from an Ensembl data file for the canine reference genome (CanFam3.1 [26],). Genes were retrieved using the gene-based method, considering a ± 100 kb window around each marker. Gene-set enrichment was performed using the gprofiler2 package [27] available in R. Candidate gene symbols were tested for over-representation across functional categories using *Homo sapiens* as the reference organism to leverage curated functional resources and orthology-supported annotations. Enrichment significance was assessed using FDR multiple-testing correction, and terms with adjusted p < 0.05 were retained as significant.

## Results

### Population structure

Table 1 summarizes the genomic inbreeding coefficients for the Doberman Pinscher population. The average inbreeding for the first four metrics was the same (0.04), with values ranging from moderate negative to high positive inbreeding within the population. The metric with the highest inbreeding was the F_ROH_ metric (0.42), with the segments of 8–16Mb presenting the highest contribution to the value (0.12). In resume, The ROH-based estimator showed a higher numerical value than the allele-frequency-based metrics because F_ROH_ measures the proportion of the autosomal genome covered by ROH segments, whereas F_GRM_, F_HOM1_, F_HOM2_, and F_UNI_ are scaled relative to allele-frequency expectations. Therefore, these estimators capture related but non-identical aspects of genomic homozygosity and are not expected to be on the same numerical scale.

**Table 1:** Summary of different inbreeding coefficient metrics in the Doberman Pinscher population.

Inbreeding	Min	Max	Mean	SD	Median	Q1	Q3
F_GRM_	−0.19	0.71	0.04	0.13	0.01	−0.05	0.11
F_HOM1_	−0.20	0.51	0.04	0.09	0.04	−0.02	0.10
F_HOM2_	−0.30	0.50	0.04	0.11	0.05	−0.04	0.12
F_UNI_	−0.10	0.49	0.04	0.07	0.03	0.00	0.07
F_ROH_	0.22	0.68	0.42	0.08	0.41	0.33	0.46
F _< 2Mb_	0.02	0.11	0.05	0.01	0.03	0.03	0.03
F_2-4Mb_	0.02	0.15	0.06	0.01	0.06	0.05	0.06
F_4-8Mb_	0.04	0.20	0.10	0.02	0.10	0.08	0.11
F_8-16Mb_	0.01	0.23	0.12	0.03	0.12	0.10	0.15
F _> 16Mb_	0.00	0.41	0.09	0.06	0.09	0.04	0.13

Min, minimum individual inbreeding coefficient; Max, maximum individual inbreeding coefficient; Mean, average of the inbreeding coefficient; SD, standard deviation of the inbreeding coefficients; Median: median value; Q1: first quartile, corresponding to the 25^th^ percentile; Q3: third quartile, corresponding to the 75^th^ percentile; F_GRM_, inbreeding coefficient based on the genotype additive variance; F_HOM1_, inbreeding coefficient based on the homozygous genotypes observed and expected; F_HOM2_, inbreeding coefficient based on homozygous genotypes; F_UNI_, inbreeding coefficient based on the correlation between uniting gametes; F_ROH_, inbreeding coefficient based on the length of the ROH’s and the total length of the autosomal genome.

Fig 1 shows the correlation among the different metrics. In general, the correlation ranged from low to high and presented positive and negative correlations. Strong and positive correlations were found between F_HOM1_, F_HOM2_, F_UNI_, and F_ROH_. Also, the metrics F_HOM1_, F_HOM2_, and F_ROH_ had positive and high correlation with the long segments of ROH (F_8-16Mb_ and F_>16Mb_). Weak to moderate negative correlations were observed with the metrics of F_GRM_ and the other metrics, with F_UNI_ being the only metric to have a strong and positive correlation with F_GRM_ (0.63).

**Fig 1:**
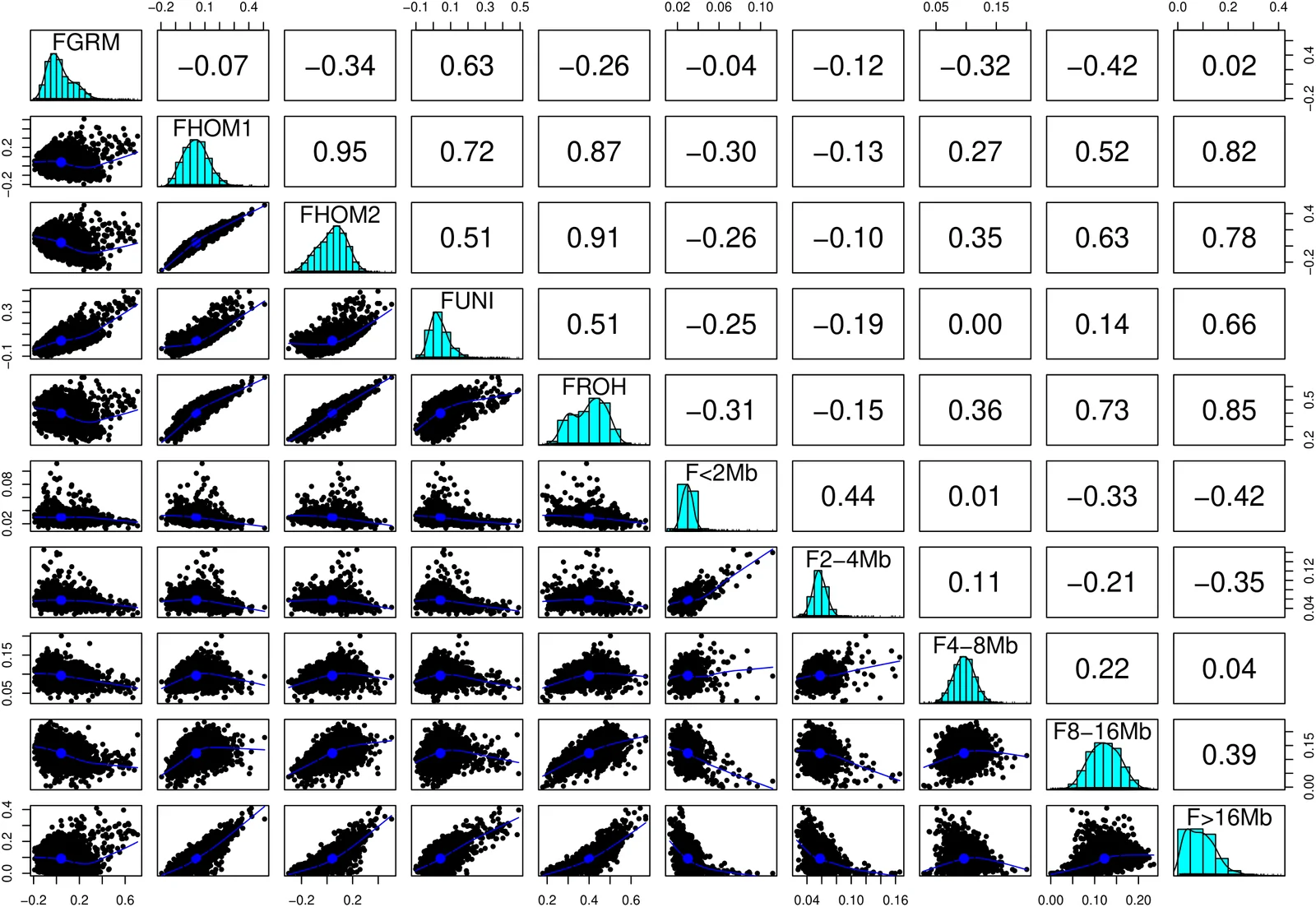
Distribution and pairwise correlations among genomic inbreeding coefficients and ROH-based inbreeding estimates. The diagonal panels show the distribution of each genomic inbreeding coefficient, the lower diagonal panels show pairwise scatterplots with fitted trend lines, and the upper diagonal panels show pairwise correlation coefficients. F_GRM_: inbreeding coefficient based on the genotype additive variance; F_HOM1_: inbreeding coefficient based on the homozygous genotypes observed and expected; F_HOM2_: inbreeding coefficient based on homozygous genotypes; F_UNI_: inbreeding coefficient based on the correlation between uniting gametes; F_ROH_: inbreeding coefficient based on the length of the ROH’s and the total length of the autosomal genome.

S3 Fig shows the LD decay and the plot of the first three PCAs for the Doberman Pinscher population. On average, the LD between the markers was higher at shorter distances (LD = 0.64 at 0.01kb) and decreased with increasing distance evaluated (LD = 0.20 at 1,000 kb). In the PCAs, the first three PCAs were responsible for explaining 7.35% of the variation among individuals. Fig 2 shows the distribution of the ROH per chromosome and the percentage of the chromosome covered by ROH.

**Fig 2:**
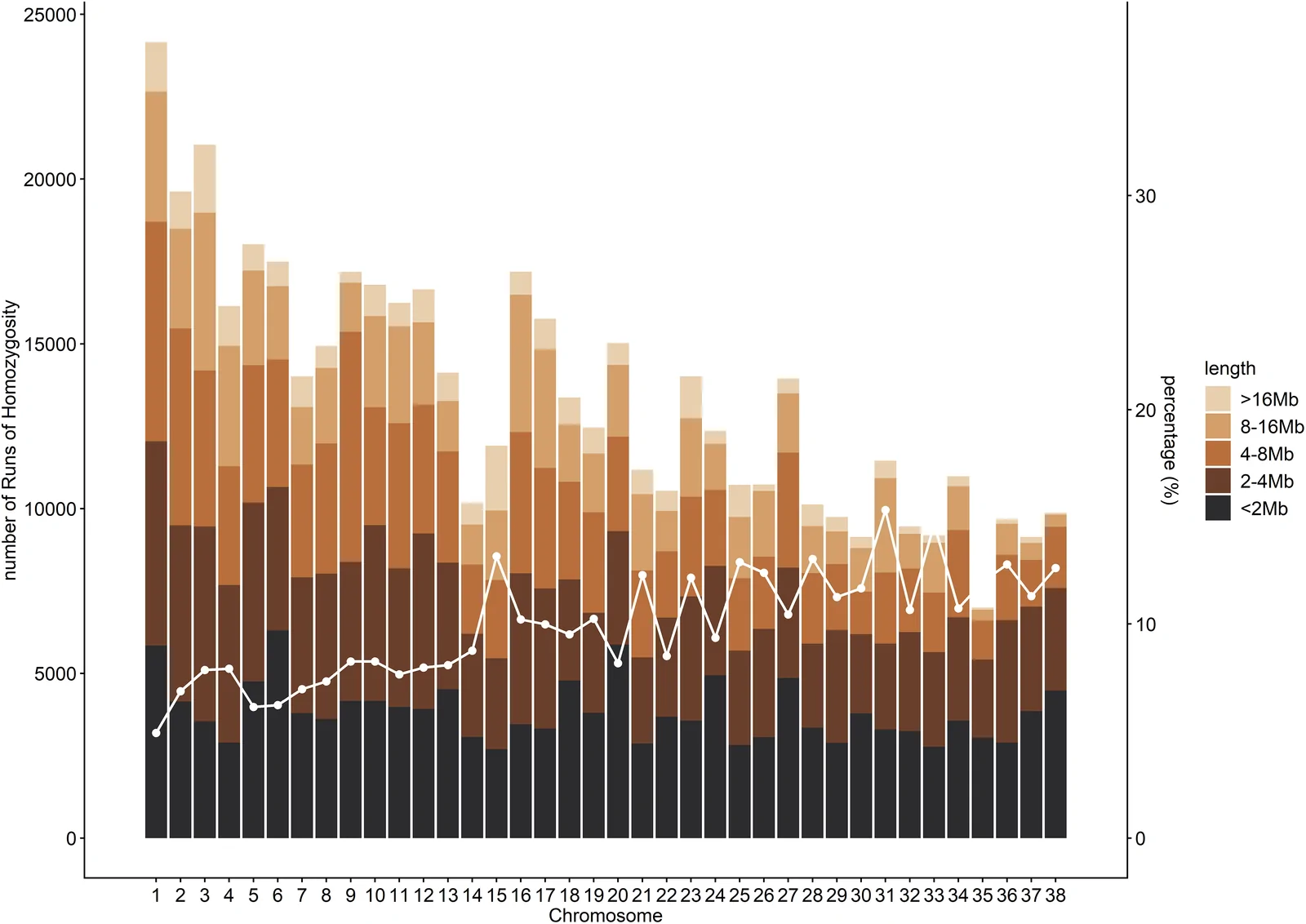
Distribution of Runs of Homozygosity per chromosome and length, and percentage of the chromosome covered by Runs of Homozygosity in the Doberman Pinscher population.

The chromosome with the highest number of ROHs was chromosome 1 (CFA1), and the chromosome with the highest coverage by the ROHs was CFA31 (~15.65%). The majority of ROHs in the population correspond to segments of <2Mb (~28.5%) and 2–4Mb (~27.8%). Long segments of ROH (≥8Mb) corresponding to ~20.6% of the total ROHs identified in the Doberman population, with ~5.4% of the segments longer than 16Mb.

#### Intrapopulation selection signature methods.

Fig 3 shows the Manhattan plots for the three intrapopulation selection-signature analyses performed in the Doberman Pinscher population, along with a Venn diagram showing the significant markers identified by each method and their overlap.

**Fig 3:**
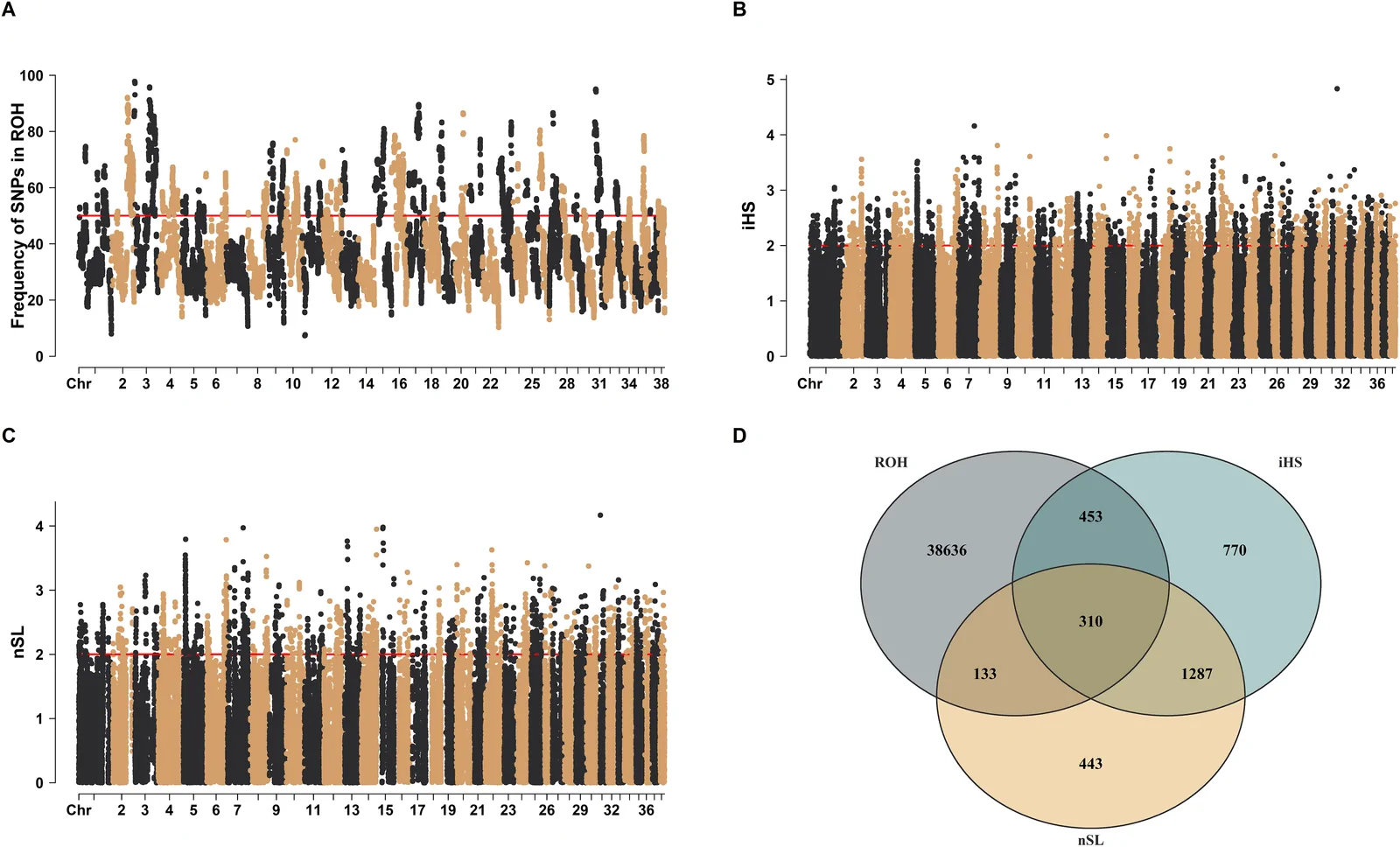
Genome-wide distribution and overlap of ROH islands and haplotype-based signatures of selection in Doberman Pinscher. (A) Frequency of SNPs within runs of homozygosity (ROH) across the genome. A 50% occurrence threshold was used to define ROH islands. (B) Manhattan plot of the integrated haplotype score (iHS) in the Doberman population. Points represent absolute normalized iHS values (|iHS|) for each SNP; loci with |iHS| > 2 were considered candidates for recent or ongoing positive selection. (C) Manhattan plot of the normalized number of segregating sites by length (nSL) values. Points represent absolute normalized nSL values (|nSL|) for each SNP; loci with |nSL| > 2 were considered candidates for recent positive selection based on unusually long haplotypes. (D) Venn diagram showing the overlap among candidate loci identified by ROH islands, iHS, and nSL analyses in the Doberman population.

In the ROH analysis, 39,512 markers were identified and located within ROH segments in more than 50% of the individuals. These markers were distributed across most chromosomes, except for CFA7, CFA14, CFA22, CFA32, and CFA35. For instance, 580 markers were mainly concentrated on CFA2, CFA3, and CFA31 (chromosomes that showed ROHs with highest concentration in the population). In the iHS analysis, 2,820 markers were identified with signs of recent ongoing selection (|iHS| > 2) in all autosomal chromosomes. From these, 763 markers were also identified in an ROH island. Regarding the sNL, the number of candidate markers for recent selection was 2,173. These markers were found across all autosomal chromosomes, and the number of markers in common with those in ROHs islands was 443, and with iHS was 1,597.

In total, 310 markers were shared across all three methods, providing strong evidence of selection signatures in these regions. These markers were located near genomic regions containing 279 annotated genes, including protein-coding genes (176), long non-coding RNAs (74), microRNAs (3), pseudogenes (4), small nucleolar RNAs (4), and small nuclear RNAs (18). Table 2 summarizes a subset of the genes located near markers identified by all three methods. The complete gene list is provided in S1 Table.

**Table 2:** Candidate genes located near genomic markers identified by all three methods of intrapopulation signatures of selection.

CHR	START	END	ENSEMBL GENE ID	GENE NAME
1	92,898,274	92,978,260	ENSCAFG00000002067	*SLC1A1*
1	93,142,635	93,435,997	ENSCAFG00000002102	*JAK2*
5	14,503,902	14,509,113	ENSCAFG00000029265	*THY1*
5	14,525,797	14,549,291	ENSCAFG00000012042	*USP2*
5	14,564,935	14,705,257	ENSCAFG00000012187	*NLRX1*
5	14,567,238	14,598,149	ENSCAFG00000012079	*MCAM*
5	14,607,013	14,676,956	ENSCAFG00000012100	*CBL*
5	14,795,750	14,807,262	ENSCAFG00000012126	*HYOU1*
6	73,823,826	74,634,764	ENSCAFG00000020411	*NEGR1*
8	59,484,328	59,612,750	ENSCAFG00000017343	*KCNK10*
8	67,622,708	67,714,404	ENSCAFG00000017809	*BCL11B*
12	1,341,191	1,354,584	ENSCAFG00000000669	*EHMT2*
12	1,453,260	1,509,749	ENSCAFG00000000751	*TNXB*
12	1,515,503	1,525,110	ENSCAFG00000000761	*ATF6B*
18	47,635,788	47,912,117	ENSCAFG00000010458	*SHANK2*
31	22,248,092	22,289,802	ENSCAFG00000024758	*ADAMTS5*
33	7,266,685	7,418,106	ENSCAFG00000009506	*ABI3 BP*

CHR: canine chromosome (CFA); START and END: genomic coordinates (bp) based on the CanFam3.1 reference genome; ENSEMBL GENE ID: Ensembl gene identifier; GENE NAME: official gene symbol.

Table 3 shows the gene ontology terms enriched for biological process, cellular components, and molecular functions associated with the candidate genes located near genomic markers identified by all three methods of intrapopulation signatures of selection.

**Table 3:** Gene Ontology terms enriched among genomic regions shared by all intrapopulation selection-signature methods.

Gene Ontology ID	name	p-value
*Biological Process*		
GO:0048731	system development	0.002
GO:0007275	multicellular organism development	0.003
GO:0048856	anatomical structure development	0.005
GO:0048666	neuron development	0.005
GO:0032501	multicellular organismal process	0.005
GO:0030182	neuron differentiation	0.005
GO:0022008	neurogenesis	0.006
GO:0048699	generation of neurons	0.008
GO:0032502	developmental process	0.008
GO:0065007	biological regulation	0.015
GO:0007399	nervous system development	0.020
GO:0048513	animal organ development	0.048
*Cellular Component*		
GO:0098590	plasma membrane region	0.000
GO:0016324	apical plasma membrane	0.000
GO:0048786	presynaptic active zone	0.000
GO:0045177	apical part of cell	0.001
GO:0030427	site of polarized growth	0.001
GO:0071944	cell periphery	0.006
GO:0098552	side of membrane	0.006
GO:0030426	growth cone	0.006
GO:0150034	distal axon	0.024
GO:0005886	plasma membrane	0.024
GO:0016020	membrane	0.024
GO:0005783	endoplasmic reticulum	0.027
GO:0070161	anchoring junction	0.027
GO:0071782	endoplasmic reticulum tubular network	0.027
GO:0098793	presynapse	0.027
GO:0032584	growth cone membrane	0.031
GO:0098984	neuron to neuron synapse	0.036
GO:0098794	postsynapse	0.040
GO:0012505	endomembrane system	0.040
*Molecular Function*		
GO:0005515	protein binding	0.000

ID: Gene Ontology (GO) identifier; name: GO term description; p-value: enrichment test p-value for over-representation of the term among the candidate genes.

Functional enrichment of genes located near markers shared across all intrapopulation selection-signature methods revealed an over-representation of developmental pathways, particularly related to neurodevelopment. The most significant biological-process terms were related to system and organismal development, including neuron development, differentiation, and neurogenesis (p ≤ 0.020). Consistently, enriched cellular-component terms highlighted plasma-membrane and neuron-specific structures, including presynaptic active zone, growth cone, distal axon, and synaptic compartments (p ≤ 0.040). At the molecular-function level, protein binding was the predominant enriched term (p < 0.001).

#### Interpopulation selection signature methods.

Fig 4 shows the Manhattan plot for the FST and cross-population analysis comparing the Doberman population to the Labrador Retriever population, along with the Veen diagram comparing the significant markers identified in each one of the analyses.

**Fig 4:**
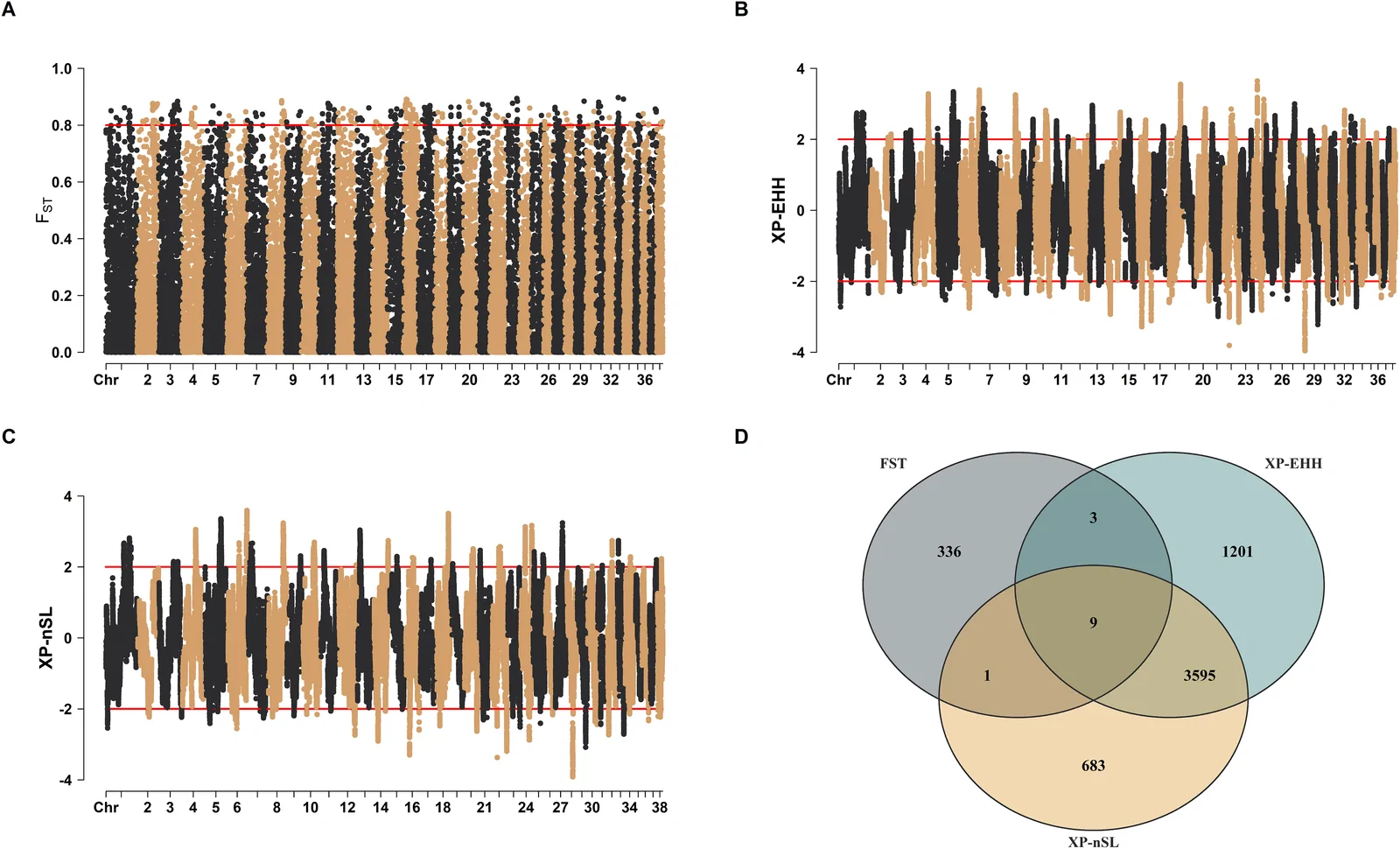
Genome-wide distribution and overlap of interpopulation selection signatures (FST, XP-EHH, and XP-nSL) between the Doberman Pinscher and the Labrador Retriever. (A) Genome-wide distribution of FST between Doberman and Labrador populations. SNPs within the upper 20% of the empirical FST distribution (≥80th percentile) were considered candidates for population-differentiated selection. (B) Cross-population extended haplotype homozygosity (XP-EHH) comparing Doberman (target) and Labrador (reference) populations. Horizontal red lines at XP-EHH = +2 and XP-EHH = −2 denote thresholds for strong population-specific selection. (C) Signed Cross-Population Number of Segregating Sites by Length (XP-nSL) analysis contrasting Doberman and Labrador populations. Horizontal red lines at XP-nSL = +2 and XP-nSL = −2 indicate thresholds for strong population-specific selection. (D) Venn diagram showing the overlap among inter-population selection signals identified using FST, XP-EHH, and XP-nSL analyses comparing Doberman (target) and Labrador (reference) populations.

For the FST analysis, 349 markers showed a high differentiation among the populations. These markers are present in all autosomal chromosomes, with CFA16 (32), CFA3 (28), CFA2 (25), and CFA11 (35) being the chromosomes that present the highest number of markers exceeding the threshold. For the XP-EHH, 3,101 markers had a differentiation from the reference population (high positive values). These markers were present in almost all autosomal chromosomes, except in CFA26 and CFA28. Ten markers were found in common among the XP-EHH and the FST metric. For the XP-nSL method, 2,832 markers showed a high differentiation from the reference population. These markers are distributed along almost all autosomal chromosomes except CFA2, CFA23, CFA26, CFA28, and CFA36. The number of markers in common between the XP-nSL method and FST was 8, and between XP-nSL and XP-EHH was 2,441.

Nine markers were found to be common across all three methods. These markers are located close to regions that encode 13 genes, as shown in Table 4. From these, 9 were protein-coding and 4 long non-coding RNAs (lncRNAs).

**Table 4:** Candidate genes near genomic markers identified by all three methods of interpopulation signatures of selection.

CHR	START POS	END POS	GENE ID	GENE NAME	GENE BIOTYPE
1	93,846,269	93,957,359	ENSCAFG00000002131	*ERMP1*	Protein coding
1	94,096,954	94,103,734	ENSCAFG00000002137		Protein coding
6	73,823,826	74,634,764	ENSCAFG00000020411	*NEGR1*	Protein coding
8	59,484,328	59,612,750	ENSCAFG00000017343	*KCNK10*	Protein coding
8	59,565,623	59,565,877	ENSCAFG00000041412		Protein coding
8	59,658,365	59,697,318	ENSCAFG00000017354	*SPATA7*	Protein coding
8	59,708,972	59,782,459	ENSCAFG00000017377	*PTPN21*	Protein coding
8	60,260,126	60,275,034	ENSCAFG00000043039		lncRNA
8	60,275,952	60,277,947	ENSCAFG00000047705		lncRNA
8	60,288,179	60,289,810	ENSCAFG00000048045		lncRNA
8	60,331,172	60,605,478	ENSCAFG00000017486		Protein coding
15	32,647,472	32,647,762	ENSCAFG00000006153	*BTG1*	Protein coding
15	32,657,752	32,666,640	ENSCAFG00000046390		lncRNA

CHR: canine chromosome (CFA); START and END: genomic coordinates (bp) based on the CanFam3.1 reference genome; ENSEMBL GENE ID: Ensembl gene identifier; GENE NAME: official gene symbol; GENE BIOTYPE: gene classification based on the type of RNA transcript produced.

Most markers mapped to a single region on CFA8 (~59.48–60.61 Mb), which included multiple protein-coding genes (*KCNK10*, *SPATA7*, *PTPN21*) and several nearby lncRNAs. Additional markers were detected on CFA1 (*ERMP1* and one unannotated protein-coding locus), CFA6 (*NEGR1*), and CFA15 (*BTG1* and one lncRNA).

## Discussion

### Population structure

The main goal of this study was to characterize population structure and identify signatures of selection in Doberman Pinschers using multiple complementary approaches. Across the first four genomic inbreeding coefficients used in this study, mean inbreeding was similar (0.04; Table 1). However, as the methods capture different aspects of autozygosity and are scaled relative to allele-frequency expectations, distinct ranges (including the presence of both negative and positive values) were identified for the different metrics. For instance, the F_GRM_ quantifies an individual’s homozygosity relative to the sample mean using allele frequencies to define expected genotype variance, such that positive values indicate individuals that are more homozygous (i.e., more related) than the population average, and negative values indicate less homozygosity than expected [13]. The homozygosity-based estimators (F_HOM1_ and F_HOM2_) summarize deviations in observed homozygosity from the expectation under Hardy–Weinberg proportions given the population allele frequencies, thereby reflecting excess homozygosity consistent with identity-by-descent. In this context, positive values indicate excess homozygosity, whereas negative values indicate an excess of heterozygosity relative to expectation [14,15]. The F_UNI_ aims to measure autozygosity with reduced dependence on allele frequency, with the scaling focusing on the correlation between the two gametes that formed the individual; i.e., positive values indicate more correlated gametes (greater autozygosity), whereas negative values indicate less correlation [15,28].

Overall, frequency-based estimators (F_GRM_, F_HOM1_, F_HOM2_, F_UNI_) showed comparable means and included both negative and positive values (Table 1), consistent with their dependence on allele-frequency centering within the study sample. In contrast, ROH-based inbreeding was substantially higher (mean F_ROH_ = 0.42; range 0.22–0.68), indicating extensive autozygosity in this population. An advantage of the ROH method is that it measures the proportion of the genome that is homozygous and, by grouping ROH by length, gives an estimate of when inbreeding occurred in the population [29]. This feature makes the method useful for population management, because not all inbreeding is harmful [30,31]. Knowing when inbreeding events occurred helps reconstruct the population’s history. Partitioning F_ROH_ by ROH length showed that most autozygosity was attributed to medium-to-long segments (4–16 Mb), consistent with recent inbreeding, whereas the > 16 Mb class exhibited high variability (maximum 0.41), suggesting that a subset of individuals experienced very recent consanguineous matings.

Across metrics, the strong concordance between F_HOM_-based measures and F_ROH_ indicates that the main inbreeding signal is and not driven by a single estimator (Fig 1). An interesting point to observe is that most metrics have a high correlation with the more recent types of inbreeding (F_8-16Mb_ and F_>16Mb_). This suggests that, in this population, the majority of the allele-frequency drive estimator captures more recent inbreeding than older events in the population. By contrast, weaker or negative associations between F_GRM_ and other estimators were observed. This might be due to the greater sensitivity of GRM-based inbreeding to allele-frequency centering and the chosen reference frequencies, which can shift the scale (and occasionally the sign) in structured or relatively homogeneous samples [32,33].

Recent inbreeding events in the population are also evident in the distribution of ROH across the genome (Fig 2). Long segments of homozygosity were detected in all autosomal chromosomes evaluated, corresponding to around 20.6% of the ROHs identified in the genome of the Doberman Pinscher population. Based on the length of the segments, it is estimated that consanguineous mating has happened in the population around 3–5 generations ago [29,34].

#### Intrapopulation selection signatures methods.

We used three complementary methods to detect within-population signatures of selection in the Doberman Pinscher (Fig 3). First, ROH islands (Fig 3A) highlight genomic regions that are repeatedly homozygous across many individuals. Such patterns can emerge when strong and/or sustained selection increases the frequency of shared haplotypes, thereby reducing local genetic diversity [35]. By contrast, iHS (Fig 3B) is tailored to detect recent, incomplete selective sweeps. It does so by comparing haplotype homozygosity surrounding the ancestral versus derived allele at each SNP [20]. Finally, nSL (Fig 3C) is conceptually similar to iHS, but it measures haplotype length using the number of segregating sites rather than genetic distance. This reduces sensitivity to recombination-rate heterogeneity and can improve performance in regions where recombination varies across the genome [36]. Taken together, these approaches provide evidence across different time scales and genomic contexts: ROH islands are more consistent with longer-term or recurrent selection that increases autozygosity, while iHS and nSL are better at capturing sweeps that are still in progress and have not reached fixation.

Across all three methods, we found 310 SNPs that overlapped between ROH islands, iHS, and nSL, providing convergent support for loci under selection in this population (Fig 3D). This three-way overlap is notable because each method relies on different information (autozygosity versus haplotype structure) and different null expectations. Concordant signals are therefore less likely to reflect method-specific artifacts and more likely to represent true targets of selection or tightly linked variants. Genes near these shared markers point to biological themes involving extracellular matrix (ECM) remodeling, cellular stress responses, and vascular/cardiac biology, with additional connections to neurodevelopmental processes. These categories are plausible in Doberman Pinschers, given the breed’s history of intense artificial selection and the importance of cardiovascular and connective-tissue traits to breed health and performance [1].

Several overlapping candidates are consistent with an ECM and fibrosis-related signal. The *THY1* gene encodes a cell-surface glycoprotein involved in cell adhesion and stromal-cell biology, with established roles in wound healing and fibrotic remodeling, and it is widely used as a marker for particular fibroblast subpopulations across tissues [37–39]. In cardiac settings, *THY1*-positive fibroblast populations have been associated with fibrosis and reduced contractile function in experimental and translational work [40]. The *ADAMTS5* gene, which encodes a secreted metalloprotease (aggrecanase-2), cleaves ECM proteoglycans and is best known for its role in cartilage turnover and osteoarthritis-related degeneration [41,42]. While its strongest associations are musculoskeletal, *ADAMTS*-family proteases more broadly contribute to ECM remodeling, making *ADAMTS5* relevant if selection is acting on matrix dynamics across tissues [43]. The *ABI3 BP* encodes an ECM-associated protein expressed in cardiovascular tissues and has been linked to cell–ECM interactions and remodeling phenotypes, supporting a potential role in structural maintenance and cardiovascular function [44]. The *TNXB* gene, a tenascin-family ECM gene, is also consistent with selection influencing connective-tissue architecture; although its direct link to specific Doberman Pinscher traits may be indirect, tenascin biology is often discussed in relation to tissue integrity and cardiovascular/connective-tissue pathology [45,46].

A second set of overlapping candidates fits stress-response and protein homeostasis pathways, which are repeatedly implicated in cardiac disease and hypertrophic remodeling. The *JAK2* gene is a key component of JAK/STAT signaling and is broadly involved in inflammatory and stress signaling, with extensive experimental support linking this pathway to cardiac hypertrophy and heart failure biology [47]. The genes *HYOU1* and *ATF6B* map to the unfolded protein response and endoplasmic reticulum stress pathways, which are activated under ischemic and hypertrophic stress and can influence cardiomyocyte survival and remodeling outcomes [48]. In line with this theme, *USP2* (a deubiquitinase) and *CBL* (an E3 ubiquitin ligase) connect to ubiquitin-mediated regulation of signaling and proteostasis; both have been reported in studies involving oxidative stress and hypertrophic or heart failure–related phenotypes, depending on the system and context [49,50]. The *NLRX1* gene, a mitochondrial innate immune receptor, further supports a mitochondria–stress axis, as it has been implicated in regulating mitochondrial function and injury responses, including potentially protective roles in ischemia/reperfusion and other stress-related settings [51].

Finally, overlapping loci near genes with vascular and endothelial relevance may indicate selection acting on pathways shaping vascular biology and fibrosis. The *MCAM* (*CD146*) gene is commonly described as an endothelial/vascular marker and has been linked to endothelial processes that intersect with fibrotic remodeling and vascular function, and it has also been explored as a biomarker or association signal in some cardiovascular studies [52,53]. Overall, the shared signals across ROH islands, iHS, and nSL suggest that selection in Doberman Pinschers may have targeted (or co-selected via linkage) genomic regions involved in ECM remodeling and cellular stress pathways, with additional contributions from vascular and neurodevelopment-related genes. These results provide coherent candidates for follow-up, including fine-mapping of overlapping intervals, deeper characterization of local haplotype structure, and integration with phenotype data (e.g., cardiac traits) to test whether these signals translate into measurable, breed-relevant outcomes.

#### Interpopulation selection signatures methods.

To perform the interpopulation selection analyses, we compared the Doberman Pinscher population with a Labrador Retriever reference panel derived from Campbell et al. [23]. This reference population provided an available external comparison group for between-population analyses. However, this dataset was not designed to represent the full genetic diversity of the Labrador Retriever breed. This external reference allowed us to identify genomic regions differentiating Doberman Pinschers from the available Labrador Retriever reference population.

We applied three complementary approaches to capture population selection in Dobermans. The FST (Fig 4A) quantifies allele-frequency differentiation between breeds; elevated FST values indicate loci that diverge more than expected under neutrality and may reflect population-specific selection [54]. The XP-EHH (Fig 4B) contrasts the decay of haplotype homozygosity between populations and is particularly powerful for detecting completed or nearly completed sweeps, where selection has driven a haplotype close to fixation in one breed but not in the other [21]. The XP-nSL (Fig 4C) extends the nSL framework to a cross-population setting and leverages haplotype information while explicitly contrasting populations to detect both ongoing and near-fixed sweeps [22].

Because each metric emphasizes different aspects of the selective process, signals that agree across methods are more compelling than results from any single test. In general, FST reflects allele-frequency divergence, whereas XP-EHH and XP-nSL are more sensitive to sweep dynamics that are specific to one population, especially under strong selection when sweeps are advanced. Combining all three, therefore, provides a broader perspective on selection in the Doberman Pinscher genome across multiple evolutionary time scales. In the interpopulation analysis, we identified nine markers shared across methods (Fig 4D), mapping to regions containing 13 nearby genes. Several of these candidates are functionally consistent with pathways relevant to cellular regulation, neurobiology, and cardiovascular physiology. For example, the *BTG1* gene is involved in cell-cycle regulation and apoptosis and is widely studied in growth control [55], while the *ERMP1* gene is linked to endoplasmic-reticulum function and cellular stress responses and has also been discussed in neurobiological contexts [56,57]. The *NEGR1* gene has established roles in neuronal development and neurite arborization and has been associated with behavioral phenotypes, including anxiety- and aggression-related traits [58,59]. The *KCNK10* gene encodes a potassium channel, and variation in cardiac ion-channel genes has been implicated in canine dilated cardiomyopathy (DCM), including in Dobermans, which have high susceptibility to this condition [60–62]. In addition, the *SPATA7* gene encodes a ciliary scaffold protein required for the maintenance of the photoreceptor connecting cilium and proper protein trafficking in retinal cells [63], and the *PTPN21* gene encodes a non-receptor protein tyrosine phosphatase involved in intracellular signaling pathways that regulate cell growth and differentiation [64].

Although some candidate genes identified in this study have biological functions related to cardiac, neurological, or developmental processes, direct evidence linking variants in these genes to Doberman dilated cardiomyopathy or behavioral phenotypes is currently lacking. Previous studies have reported candidate loci for Doberman DCM [2,60,61]; however, the candidate regions detected in the present study should be interpreted as biologically plausible loci rather than confirmed disease-associated variants. Because individual-level DCM case-control status and behavioral phenotypes were not available, association analyses could not be performed. Further association studies using phenotype records, case-control datasets, or functional validation experiments are required to determine the role of these genes in Doberman health and breed-specific traits. Overall, the cross-method overlap highlights a focused set of differentiated loci with consistent evidence for signals differentiating Doberman Pinschers from the Labrador Retriever reference population selection and supports follow-up work to refine causal variants and connect these regions to breed-relevant phenotypes.

### Limitations and future directions

Certain methodological decisions in this study follow common practice for genome-wide selection scans, but there are also important limitations in how the findings should be interpreted and how candidate loci should be prioritized. Because selection statistics are continuous rather than discrete, researchers must define candidate regions using ranking-based criteria, which typically do not fit neatly into a formal hypothesis-testing framework. As a result, the specific cutoffs used to define candidate regions can meaningfully influence both the number and type of loci that are reported—especially for loci whose values fall close to the threshold.

In this study, candidate loci were identified using multiple outlier detection approaches. The ROH islands were defined as markers that occurred within an ROH in at least 50% of individuals. Haplotype-based candidates were defined using |iHS| and |nSL| > 2 for intrapopulation scans, and |XP-EHH| and |XP-nSL| ≥ 2 for cross-population scans. The ROH islands threshold used in this study has been previously reported in studies with dogs [65,66] and other populations as a practical criterion for defining ROH islands [67,68]. Regarding the other threshold, because the iHS, nSL, XP-EHH, and XP-nSL statistics are typically standardized across the genome — often within bins of allele frequency — score values are less dependent on underlying allele frequency. For that reason, a cutoff around |score| ≈ 2 is a commonly used heuristic cutoff for standardized haplotype signals [20–22]. However, the observed score distribution may not perfectly match test assumptions, so this type of cutoff should be interpreted as an outlier filter rather than a strict significance test [19,69,70].

For allele frequency differentiation, we used a stringent threshold (FST > 0.80) to prioritize markers that are highly differentiated between Doberman Pinschers and the Labrador population. This emphasizes extreme divergence consistent with near-fixation differences, but it may fail to capture more moderate patterns that could reflect polygenic selection and/or drift [71]. Finally, genotypes in this study were derived from SNP array data, which introduces uneven genome coverage and a bias towards common variants. Future work should validate key regions using imputed sequence data or whole-genome sequencing to help distinguish a single strong selective sweep from broader plateaus driven by linkage disequilibrium and/or overlapping selection signals.

Also, limitation of worth to highlight between-population analyses is the use of a single Labrador Retriever reference population. Although additional analyses were performed to describe its genomic structure, LD decay, genomic diversity, and ROH patterns, individual-level information was not available. Therefore, potential internal substructure within the Labrador Retriever reference population could not be fully assessed. As a result, selection signals detected using FST, XP-EHH, and XP-nSL should be interpreted as contrasts between Doberman Pinschers and the available Labrador Retriever reference population, rather than as definitive Doberman-specific signatures. Independent validation using additional reference populations and phenotypically characterized samples will be important to confirm these candidate regions

## Conclusion

Overall, this study shows that the Doberman Pinscher population has high genome-wide autozygosity, consistent with recent inbreeding. Interestingly, the multiple approaches used for the selection signatures analysis showed convergent candidate signals within and between populations. These overlapping signals point to a broad functional process related to cellular modeling, stress, vascular and cardiac biology, with additional connections to neurodevelopmental processes and behavior. Overall, our findings provide a coherent set of pathways and genes that can be used as targets for follow-up studies integrating phenotypes relevant to breed health and performance.

## Supporting information

S1 Fig Pearson and Spearman correlations among genomic inbreeding coefficients estimated using SNP-based methods and runs of homozygosity stratified by ROH length classes in the Doberman Pinscher population. Panel A shows Pearson correlations and Panel B shows Spearman correlations. Overall, both methods revealed similar patterns, supporting the robustness of the relationships among genomic inbreeding estimators. Strong positive correlations were observed among SNP-based estimators and between FROH and longer ROH classes.
(PDF)

S2 Fig Genomic characterization of the Labrador Retriever reference population used for between-population selection signature analyses. (PDF)

S3 Fig Linkage disequilibrium decay (right) and principal components analysis (left) of the Doberman Pinscher population. (PNG)

S1 Table List of candidate genes near genomic markers commonly detected by three intrapopulation selection signature methods. (XLSX)
