# Supplementary material for: Genomic inbreeding and selection signatures analyses in the Doberman Pinscher breed
Source: PLoS One. 2026 Oct 8;21(10):e0351912. doi: 10.1371/journal.pone.0351912 (PMC13648987; doi:10.1371/journal.pone.0351912)
Supplement: Supplementary file 1 [file pone.0351912.s001.pdf]

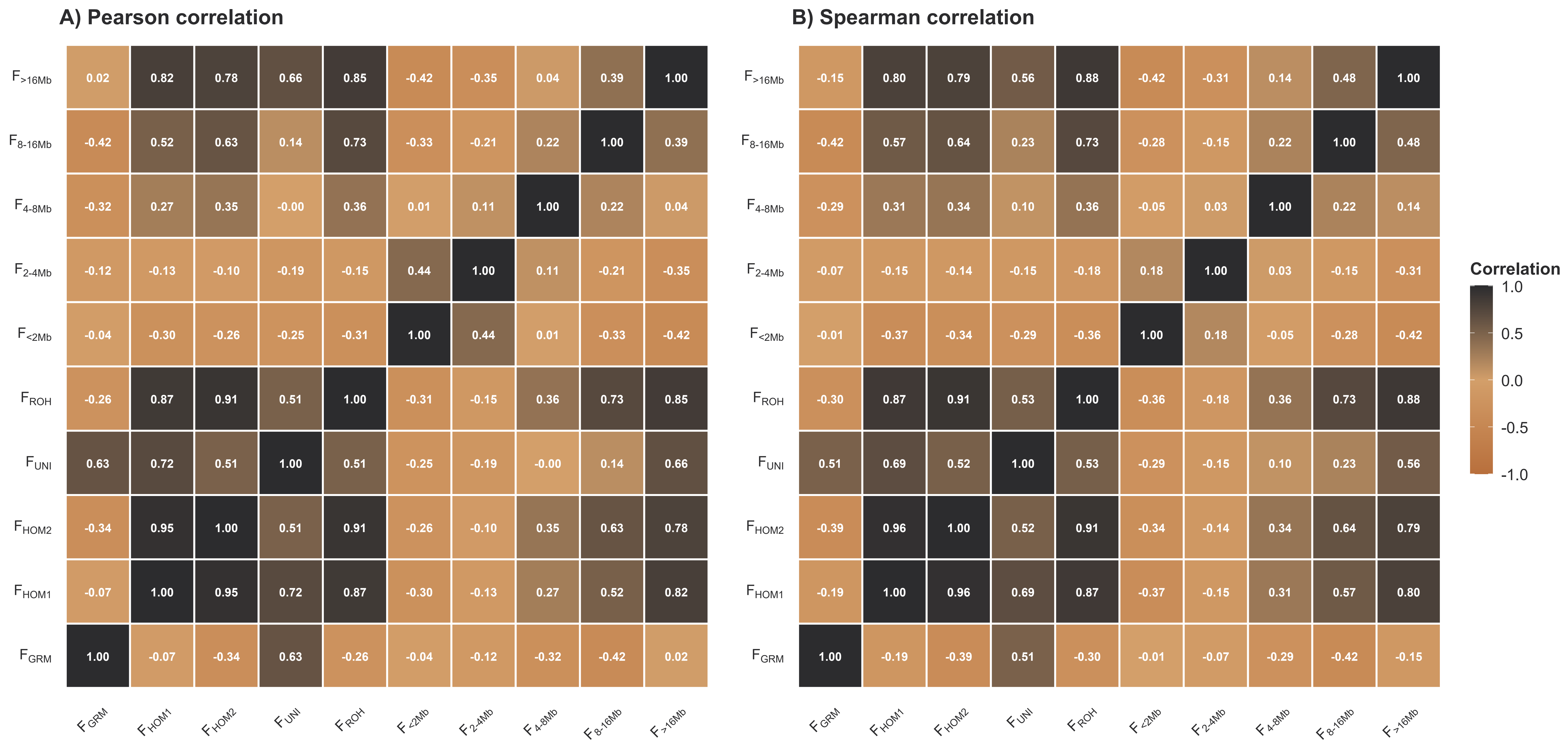

A) Pearson correlation
$F_{>16Mb}$ 0.02 0.82 0.78 0.66 0.85 -0.42 -0.35 0.04 0.39 1.00
F_{8-16Mb} -0.42 0.52 0.63 0.14 0.73 -0.33 -0.21 0.22 1.00 0.39
F_{4-8Mb} -0.32 0.27 0.35 -0.00 0.36 0.01 0.11 1.00 0.22 0.04
F_{2-4Mb} -0.12 -0.13 -0.10 -0.19 -0.15 0.44 1.00 0.11 -0.21 -0.35
$F_{<2Mb}$ -0.04 -0.30 -0.26 -0.25 -0.31 1.00 0.44 0.01 -0.33 -0.42
F_{ROH} -0.26 0.87 0.91 0.51 1.00 -0.31 -0.15 0.36 0.73 0.85
F_{UNI} 0.63 0.72 0.51 1.00 0.51 -0.25 -0.19 -0.00 0.14 0.66
F_{HOM2} -0.34 0.95 1.00 0.51 0.91 -0.26 -0.10 0.35 0.63 0.78
F_{HOM1} -0.07 1.00 0.95 0.72 0.87 -0.30 -0.13 0.27 0.52 0.82
F_{GRM} 1.00 -0.07 -0.34 0.63 -0.26 -0.04 -0.12 -0.32 -0.42 0.02
F_{GRM} F_{HOM1} F_{HOM2} F_{UNI} F_{ROH} $F_{<2Mb}$ F_{2-4Mb} F_{4-8Mb} F_{8-16Mb} $F_{>16Mb}$
B) Spearman correlation
$F_{>16Mb}$ -0.15 0.80 0.79 0.56 0.88 -0.42 -0.31 0.14 0.48 1.00
F_{8-16Mb} -0.42 0.57 0.64 0.23 0.73 -0.28 -0.15 0.22 1.00 0.48
F_{4-8Mb} -0.29 0.31 0.34 0.10 0.36 -0.05 0.03 1.00 0.22 0.14
F_{2-4Mb} -0.07 -0.15 -0.14 -0.15 -0.18 0.18 1.00 0.03 -0.15 -0.31
$F_{<2Mb}$ -0.01 -0.37 -0.34 -0.29 -0.36 1.00 0.18 -0.05 -0.28 -0.42
F_{ROH} -0.30 0.87 0.91 0.53 1.00 -0.36 -0.18 0.36 0.73 0.88
F_{UNI} 0.51 0.69 0.52 1.00 0.53 -0.29 -0.15 0.10 0.23 0.56
F_{HOM2} -0.39 0.96 1.00 0.52 0.91 -0.34 -0.14 0.34 0.64 0.79
F_{HOM1} -0.19 1.00 0.96 0.69 0.87 -0.37 -0.15 0.31 0.57 0.80
F_{GRM} 1.00 -0.19 -0.39 0.51 -0.30 -0.01 -0.07 -0.29 -0.42 -0.15
F_{GRM} F_{HOM1} F_{HOM2} F_{UNI} F_{ROH} $F_{<2Mb}$ F_{2-4Mb} F_{4-8Mb} F_{8-16Mb} $F_{>16Mb}$
Correlation
1.0
0.5
0.0
-0.5
-1.0
