# Supplementary material for: Genomic inbreeding and selection signatures analyses in the Doberman Pinscher breed
Source: PLoS One. 2026 Oct 8;21(10):e0351912. doi: 10.1371/journal.pone.0351912 (PMC13648987; doi:10.1371/journal.pone.0351912)
Supplement: Supplementary file 2 [file pone.0351912.s002.pdf]

Supplementary Material S2

Genomic characterization of the Labrador Retriever reference population used for between-population selection signature analyses

Supporting information for the manuscript: Genomic Inbreeding and Selection Signature analyses in the Doberman Pinscher Breed – Mulim et al. (2026)

Purpose of this supplementary report. The primary focus of the manuscript is the Doberman Pinscher breed. Because Labrador Retrievers were used as the reference population for between-population selection signature analyses, this supplementary report provides an exploratory genomic characterization of the available Labrador Retriever reference population.

S2.1. Rationale and scope

The main objective of the manuscript was to characterize genomic inbreeding and selection signatures in the Doberman Pinscher breed. However, because the Labrador Retriever population was used as a reference group for between-population analyses, additional exploratory analyses were conducted to describe its genomic structure, linkage disequilibrium, genomic diversity, and inbreeding patterns.

These analyses are presented as supplementary material because they support the interpretation of between-population contrasts but are not the central objective of the study. The results should therefore be interpreted as a characterization of the available Labrador Retriever reference population rather than as a comprehensive breed-wide analysis of Labrador Retriever diversity.

S2.2. Description of the reference population

The Labrador Retriever reference population was obtained from the dataset described by Campbell et al. (2016) and was used as the reference group for comparisons with Doberman Pinschers. Individual-level metadata regarding working versus show type, coat color, and geographic origin were not available for the present analyses. Therefore, potential subgroup-specific effects could not be explicitly tested.

Important interpretation note: Between-population signals reported in the main manuscript should be described as selection signatures identified in Doberman Pinschers relative to the available Labrador Retriever reference population, rather than as definitive Doberman-specific loci without independent validation.

S2.3. Principal component analysis

Principal component analysis (PCA) was performed to explore the genomic structure of the Labrador Retriever reference population. This analysis was included to provide a visual assessment of the reference population used in the between-population selection signature analyses.

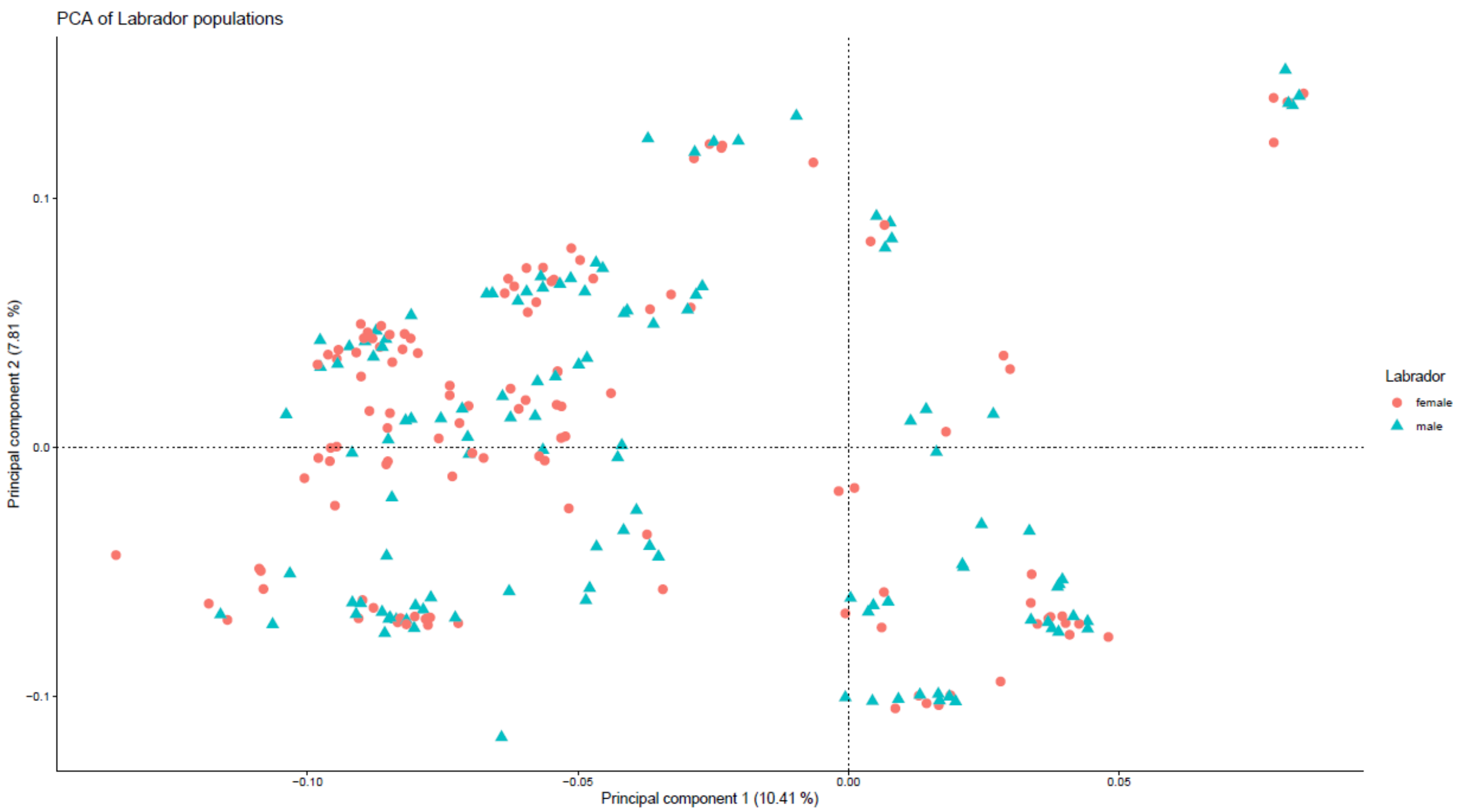

S2 Figure 1. Principal component analysis of the Labrador Retriever reference population. Individuals are colored and shaped by sex

S2.4. Linkage disequilibrium decay

Linkage disequilibrium (LD) decay was evaluated to describe haplotype structure in the Labrador Retriever reference population. This analysis provides context for the interpretation of haplotype-based between-population statistics, including XP-EHH and XP-nSL.

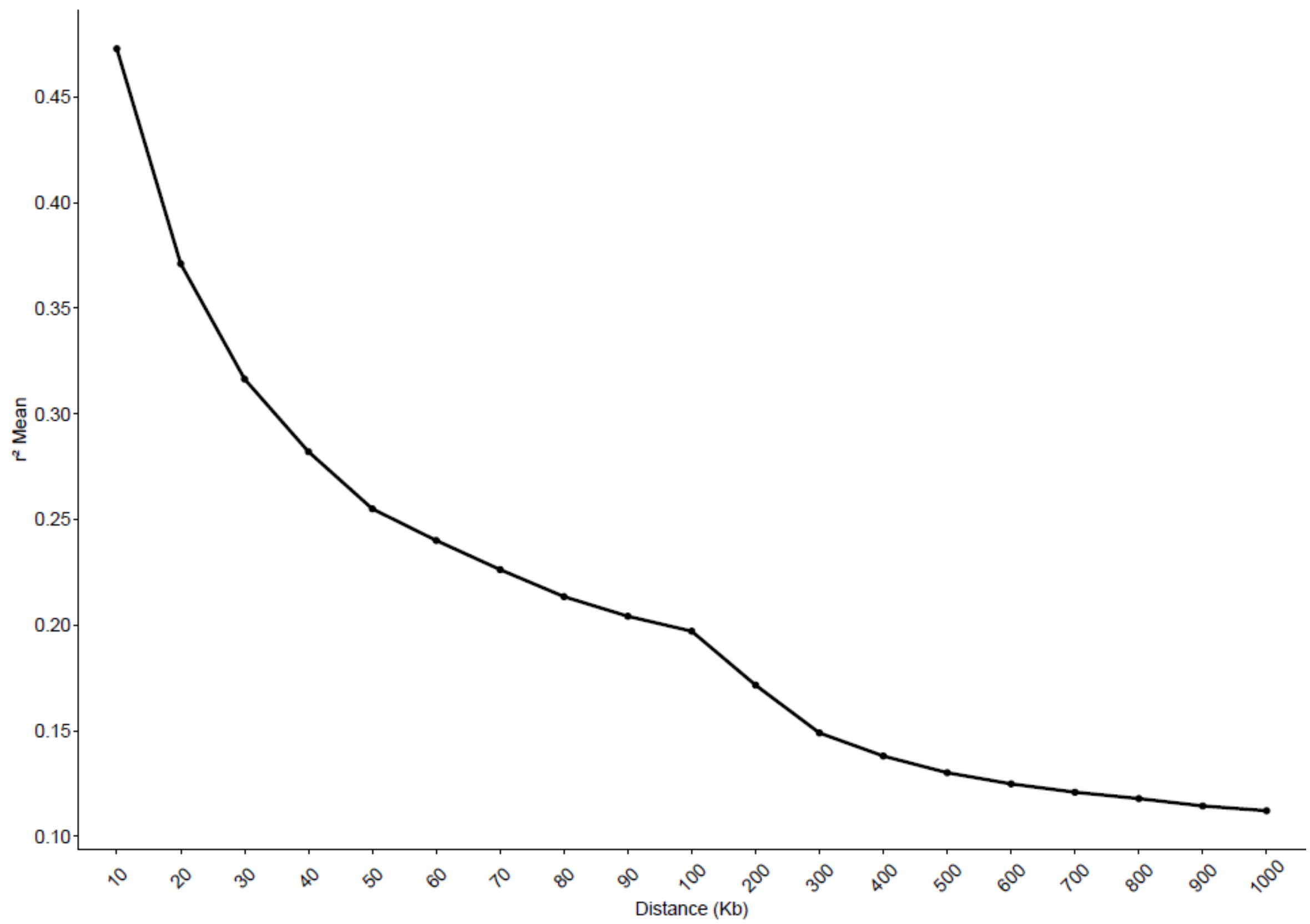

S2 Figure 2. Linkage disequilibrium decay in the Labrador Retriever reference population. LD was summarized as the decrease in average pairwise marker correlation with increasing physical distance. The observed pattern provides additional context for haplotype-based comparisons between Doberman Pinschers and the Labrador Retriever reference population.

S2.5. Genomic diversity and inbreeding coefficients

Genomic diversity and inbreeding coefficients were estimated to characterize homozygosity and autozygosity in the Labrador Retriever reference population. The evaluated metrics included SNP-based inbreeding coefficients and ROH-based inbreeding coefficients stratified by ROH length class.

S2 Table 1. Summary statistics for genomic inbreeding coefficients in the Labrador Retriever reference population.

Inbreeding coefficient	Minimum	Maximum	Mean	SD
F_{GRM}	-0.19	0.51	-0.02	0.12
F_{HOM1}	-0.18	0.45	0.01	0.09
F_{HOM2}	-0.19	0.46	0.02	0.09
F_{UNI}	-0.14	0.37	0.00	0.09
F_{ROH}	0.00	0.53	0.15	0.08
$F_{<2\ Mb}$	0.00	0.03	0.01	0.00
$F_{2\text{-}4\ Mb}$	0.00	0.06	0.02	0.01
$F_{4\text{-}8\ Mb}$	0.00	0.08	0.04	0.02
$F_{8\text{-}16\ Mb}$	0.00	0.12	0.04	0.02
$F_{>16\ Mb}$	0.00	0.34	0.04	0.05

FGRM, inbreeding coefficient based on the genomic relationship matrix; FHOM1 and FHOM2, homozygosity-based inbreeding coefficients; FUNI, inbreeding coefficient based on the correlation between uniting gametes; FROH, inbreeding coefficient based on the proportion of the autosomal genome covered by runs of homozygosity.

The observed heterozygosity in the Labrador Retriever reference population was 0.36 ± 0.03, and expected heterozygosity was 0.37 ± 0.001.

Interpretation: The mean FROH was 0.15, indicating that approximately 15% of the autosomal genome was contained within ROH segments, on average. The similarity between observed and expected heterozygosity suggests that this reference population maintained comparable observed and expected levels of heterozygosity under the evaluated marker set.

S2.6. Correlations among genomic inbreeding coefficients

Pairwise correlations among genomic inbreeding coefficients were estimated to evaluate the consistency among SNP-based and ROH-based measures of inbreeding. This analysis helps identify whether different estimators capture similar or distinct components of genomic homozygosity.

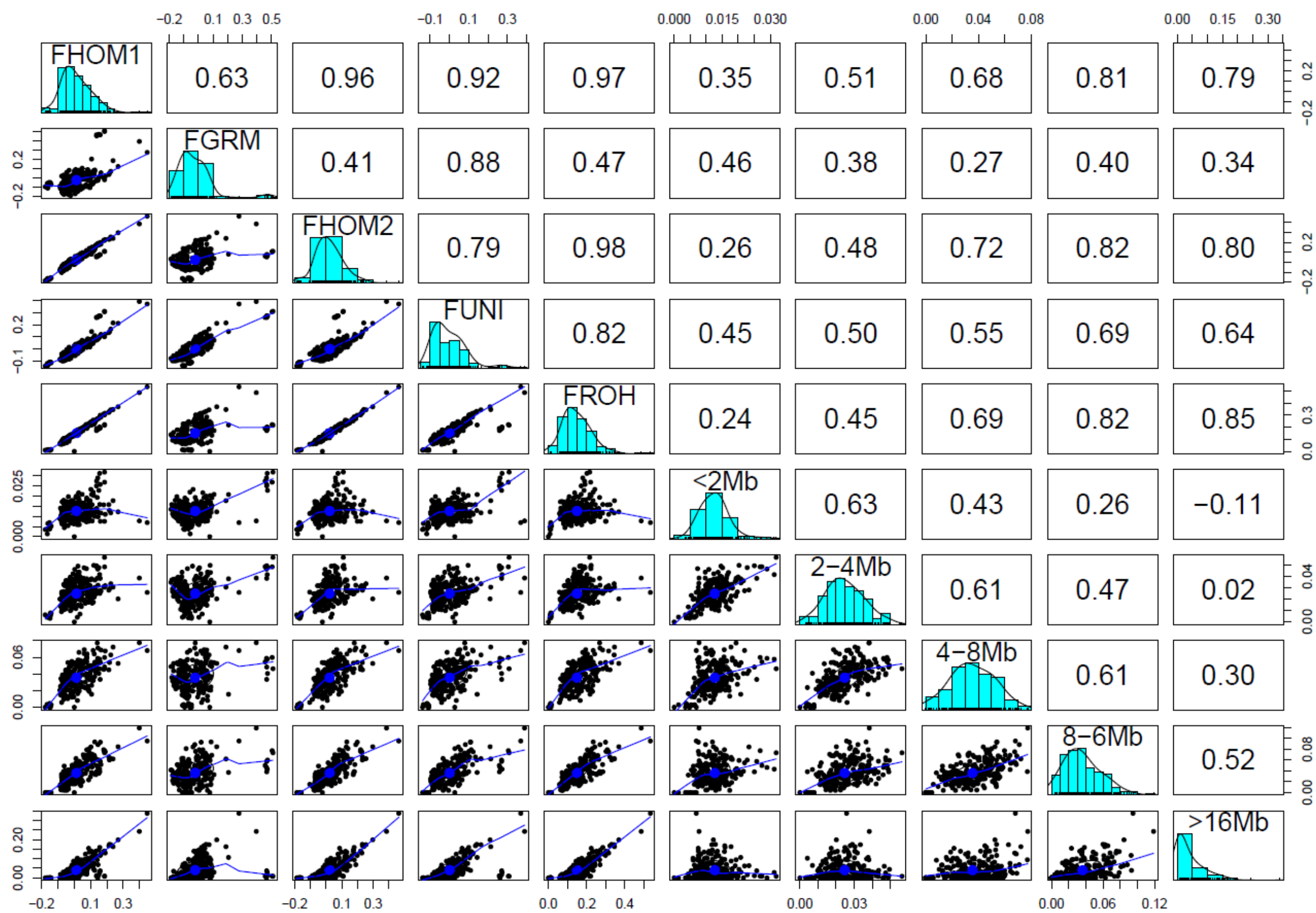

S2 Figure 3. Pairwise correlations among genomic inbreeding coefficients in the Labrador Retriever reference population. The lower diagonal shows scatterplots between pairs of inbreeding coefficients, the diagonal shows the distribution of each coefficient, and the upper diagonal shows pairwise Pearson correlation estimates.

S2.7. Distribution of ROH by chromosome and length class

The number and distribution of runs of homozygosity were summarized by chromosome and ROH length class to evaluate whether homozygosity was broadly distributed across the genome or concentrated in specific chromosomes or genomic regions.

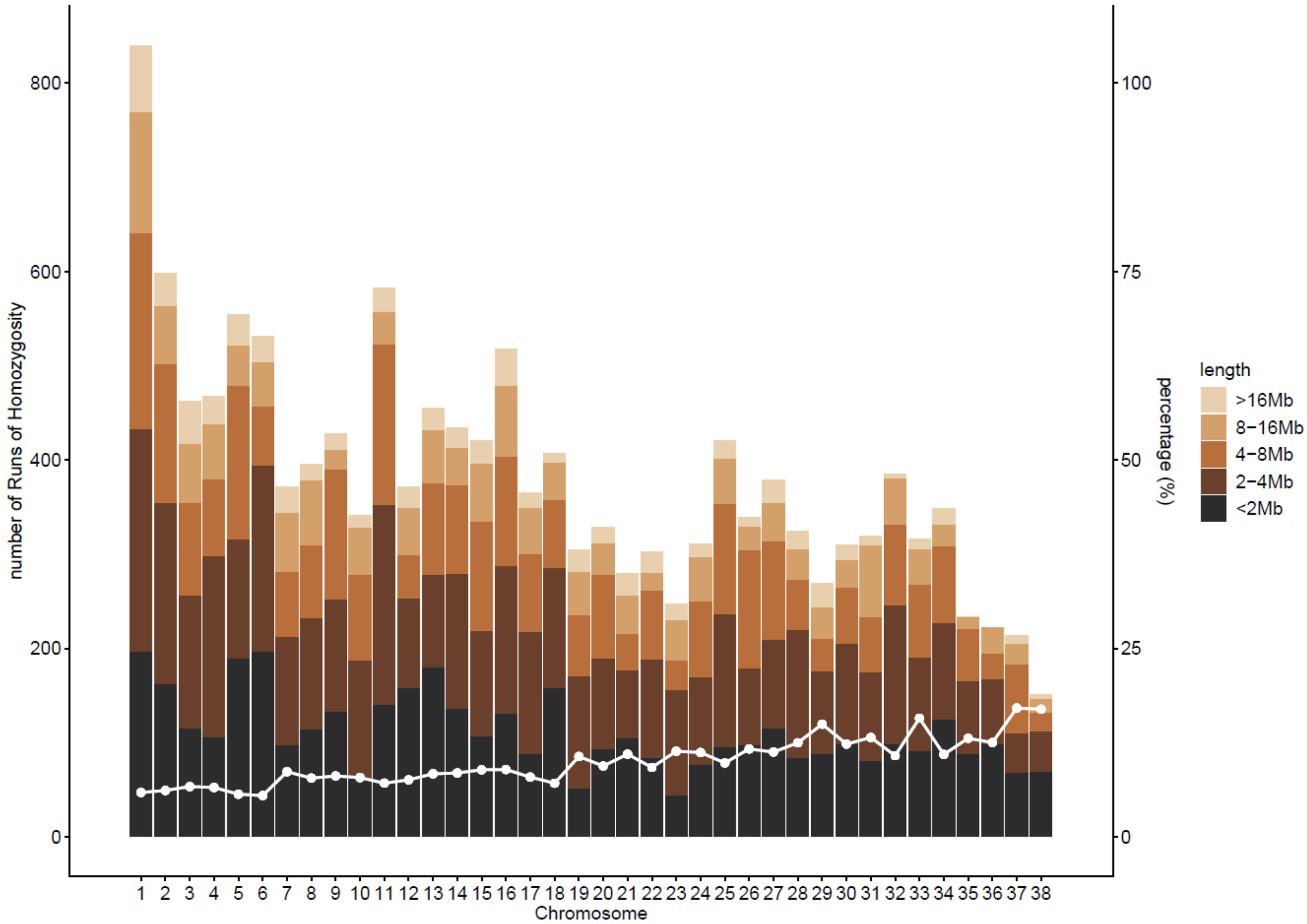

S2 Figure 4. Distribution of runs of homozygosity by chromosome and length class in the Labrador Retriever reference population. Bars represent the number of ROH segments identified on each chromosome, stratified by ROH length class. The line represents the percentage of each chromosome covered by ROH.

S2.8. ROH islands

ROH islands were evaluated to identify genomic regions with recurrent homozygosity across individuals in the Labrador Retriever reference population. A 50% SNP occurrence threshold was used to define ROH islands.

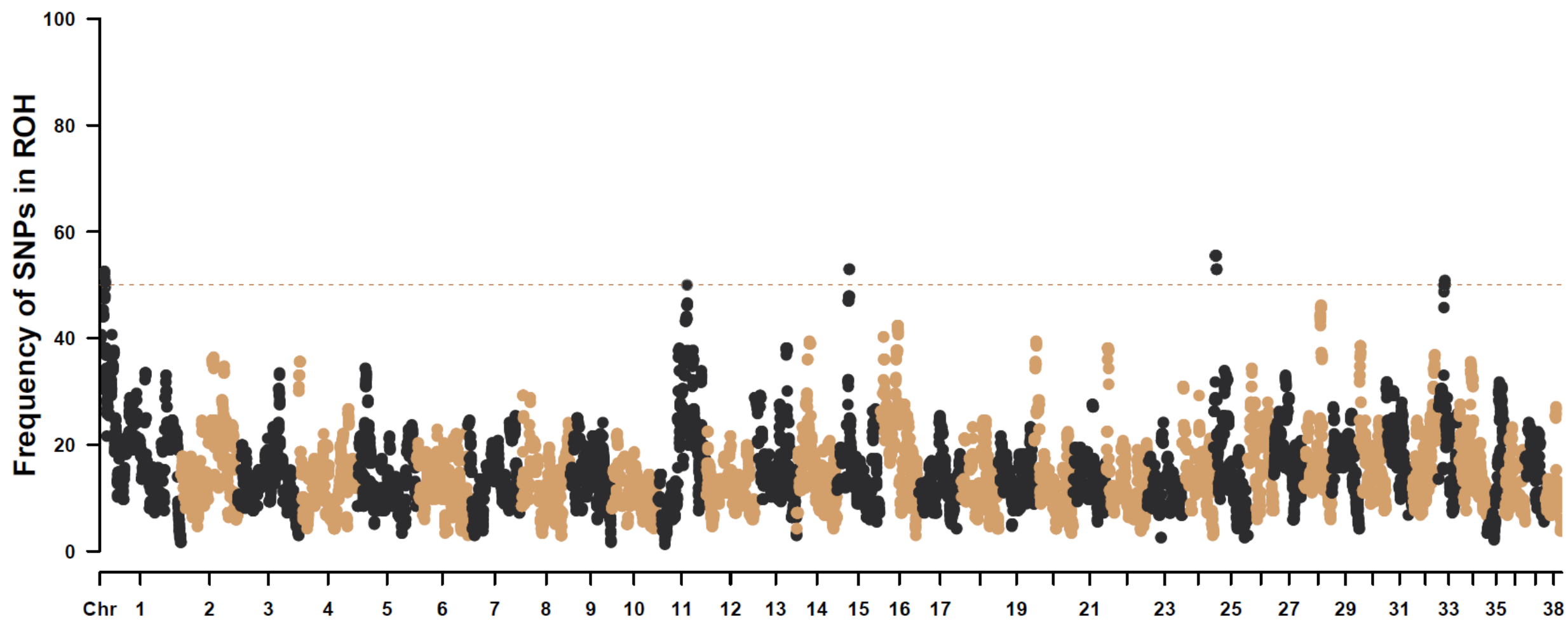

S2 Figure 5. Frequency of SNPs within runs of homozygosity across the Labrador Retriever reference genome. Each point represents the frequency with which a SNP was observed within ROH across individuals. The horizontal dashed line indicates the 50% threshold used to define ROH islands.

S2.9. Limitations of the reference population

Although these supplementary analyses provide additional information about genomic structure, LD decay, genomic diversity, and ROH patterns in the Labrador Retriever reference population, some limitations remain. Individual-level information regarding working versus show type, coat color, and geographic origin was not available. Therefore, potential internal substructure related to these factors could not be explicitly evaluated. For this reason, between-population selection signature results should be interpreted as contrasts between Doberman Pinschers and the available Labrador Retriever reference population. Independent validation using additional reference populations, larger sample sets, and phenotypically characterized dogs will be important to confirm whether the identified regions represent robust Doberman-specific selection signatures.

Reference

Campbell, C. L., Bhérer, C., Morrow, B. E., Boyko, A. R., & Auton, A. (2016). A pedigree-based map of recombination in the domestic dog genome. G3: Genes|Genomes|Genetics, 6(11), 3517–3524.
